# Supplementary figures and images for: Increased Mortality with Intermediate Ascitic Polymorphonuclear Cell Counts Amongst Patients with Cirrhosis: Time to Redefine the Care Approach
Source: Pathophysiology. 2025 Nov 11;32(4):62. doi: 10.3390/pathophysiology32040062 (PMC12641635; doi:10.3390/pathophysiology32040062)

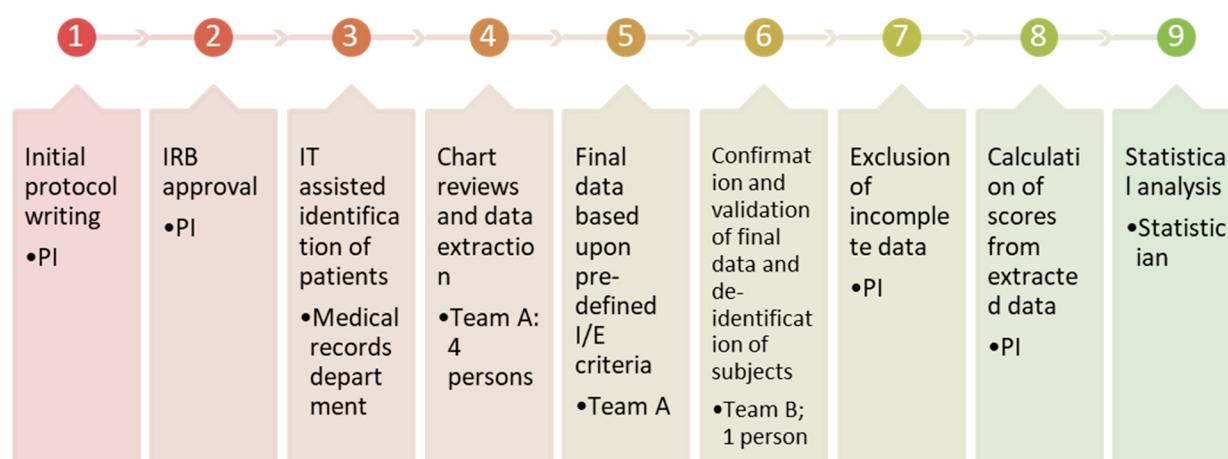

**Supplementary Figure S1:** Data extraction and handling process

Supplement: Supplementary file 1 [file pathophysiology-32-00062-s001.zip › pathophysiology-3845752-supplementary.pdf]
